# Socio-economic differences in receiving care by the over-80s in Germany and England: intensity of care needs as a moderator

**DOI:** 10.1007/s10433-025-00864-y

**Published:** 2025-06-13

**Authors:** Ursula Henz, Michael Wagner

**Affiliations:** 1 Department of Sociology, London School of Economics and Political Science, London, UK; 2 Department of Sociology and Social Psychology and Cologne Center for Ethics, Rights, Economics and Social Sciences of Health, University of Cologne, Cologne, Germany

**Keywords:** Informal care, Formal care, Unmet care need, Inequality, Care regimes, Ageing

## Abstract

The growing number of people aged 80 or older living in the community has raised concerns about meeting their care needs and about socio-economic inequalities in their care use. The study examines socio-economic status (SES) patterns in informal and formal care use, as well as unmet care needs, of people aged 80 or older living in the community in Germany and England. We propose that SES patterns in care use change with the intensity of care needs. The analyses use data from the Survey of Quality of Life and Well-Being of the Very Old in North Rhine-Westphalia and the English Longitudinal Study of Ageing. Despite the differences in the long-term care systems (LTCSs) and cultural norms around filial obligations, we find a consistent pattern of association between socio-economic status (SES) and care use for older people with only few care needs in both countries. In this group, people with a higher SES have the highest likelihood of experiencing unmet care needs. For older people with many care needs, we find country-specific SES patterns that we link to cultural differences and the design of the LTCSs. In Germany, SES is negatively associated with using informal care and positively with using formal care. In England, care use shows little SES variation for older people with many care needs. The findings underscore the importance of considering the intensity of care needs when assessing inequalities in care access.

**Supplementary Information:**

The online version contains supplementary material available at 10.1007/s10433-025-00864-y.

## Introduction

In many Western societies, the number of people aged 80 or older is increasing. The age of 80 is often seen as the beginning of a phase in the life course that is characterized by increased care needs and declining functional health (Baltes and Mayer 1999). Satisfying the care needs of the rising number of people in their 80s and older is one of the biggest challenges to ageing societies. Individual resources and the entitlement to publicly funded social care are major factors that enable care use (Andersen 1995).

Past research has observed differences in care use between SES groups (cf. scoping review by Hasseler et al. 2024). Cross-national studies have documented no or a negative association between SES and receiving informal care (Broese van Groenou et al. 2006). SES patterns of using formal care were more heterogeneous (Albertini and Pavolini 2017; Broese van Groenou et al. 2006; Floridi et al. 2021; Tenand et al. 2020).

All these studies assume a country-specific single pattern of association between SES and care use. However, this assumption overlooks that people’s options regarding their care vary with the intensity of their care needs. Applying a rational-choice approach, we argue that when people have only few care needs, they might prefer not to receive any care over the care alternatives. In contrast, when people have many care needs, the costs of foregoing care are much higher. The person’s options will be limited to the choice of either or both of formal and informal care, if the options are available.

We are going to test our proposition for England and Germany. Recent typologies of LTCSs distinguish policies that support family carers from de-familializing policies that facilitate using care services (Leitner 2003; Grages et al. 2021). The English and German LTCSs are mixed systems that include both types of policies. Both have been classified as ‘supported defamilialization through the market’ (Verbakel et al. 2023), which implies strong SES differences in formal care use in favour of the highest SES groups (Leitner 2003). However, the classification neglects the German option to transfer cash benefits to informal carers (Verbakel et al. 2023; Saraceno 2010) and the means-tested support in England. These differences might affect SES patterns in care use.

The first aim of this research is to establish whether SES is associated with informal and formal care use by people aged 80 or older in the different contexts of Germany and England. The second aim is to ascertain whether SES differences in care use change with care intensity. We analyse the care received by people aged 80 or older (‘aged 80+’ from here) living in the community. Informal care is defined as care given by family members or friends. Formal care consists of care services that are provided in a professional capacity or on a contractual basis, like home help and attending day-care centres.

Our analyses use the eighth wave of the ‘English Longitudinal Study of Ageing’ (Clemens et al. 2019) and the Survey of ‘Quality of life and well-being of the very old in North Rhine-Westphalia’ (Hansen et al. 2021). The latter is a survey of people aged 80+ in North Rhine-Westphalia (NRW), the most populous German state. Health and social care policies do not differ between NRW and Germany as a whole.

The study contributes to the literature by focusing on people aged 80+, who are not sufficiently represented in studies of receiving care despite their high care needs (Hasseler et al. 2024). Public policies increasingly try to facilitate independent living in the community for all older adults (Dorin et al. 2014; Maplethorpe et al. 2015). However, many people aged 80+ have limited access to informal care because of widowhood, spouses’ own frailty and children’s competing commitments, which increases the importance of formal care services for the oldest-old living in the community. The study analyses the social stratification of receiving care and unmet care needs, which has not been addressed for this group. We expand the explanatory framework for SES inequalities in using care by introducing the intensity of care needs as a moderator of the effects of SES. Finally, the country comparison generates evidence against which possible policy effects are examined.

## Receiving care

### Sources of care in England and Germany

For people living in the community, most care is provided by informal carers, typically spouses, children, other relatives or friends (Künemund and Hollstein 2000; Larsson and Silverstein 2004; Maplethorpe et al. 2015; Wetzstein et al. 2015). In addition, people might be able to use formal care, paid for privately or from public funds. Whether publicly funded care substitutes or complements informal care has been the subject of intensive debate (e.g. Bonsang 2009; Van Houtven and Norton 2004).

England has a tradition of formal community-based social care services, albeit means tested. Germany has moved away from its traditional family-care model (Anttonen and Sipilä 1996; Anttonen et al. 2003; Bettio and Plantenga 2004) by introducing a social care insurance with nearly universal entitlements in 1995. Despite some convergence of the two LTCS, important differences remain (Glendinnig and Igl 2009; Grages et al. 2021).

Access to healthcare is universal in both countries. In 2018, Germany and the UK each spent 1.5% of their total public expenditure on their LTCSs (Grages et al. 2021). Almost all Germans are covered by a care insurance, whereas social care is means tested in England. People with savings above £23,250 (about €25,500) do not receive any help with meeting the costs of their care (National Audit Office 2018).^1^ The local council will pay for the necessary social care of individuals with savings of no more than £14,250 (about €15,600), but it does not cover all costs (Grages et al. 2021). Most publicly financed care is provided in kind; only a minority of older people opts for a ‘personal budget’ that they can use to purchase care (Glendinning 2017). Informal carers can apply for Carers Allowance if they provide many hours of care and have low earnings (National Audit Office 2018).^2^ Older people with care needs may also receive Attendance Allowance, which is typically not passed on to informal carers (Corden et al. 2010). Financial pressures on local authorities have reduced services and tightened eligibility (AgeUK 2019; Glendinning and Igl 2009; Ismail et al. 2014). Publicly funded care has increasingly focused on high-need individuals without informal care (Comas-Herrera et al. 2010).

In Germany, individuals are assigned a ‘care level’ based on an assessment of their needs. Benefits are awarded according to the care level, regardless of financial status or the availability of family carers (Glendinning and Igl 2009; Nadash et al. 2018). Entitled individuals can choose benefits in kind, cash benefits, or both. Most beneficiaries opt for cash benefits. They can use these in any way they wish but it is assumed that most beneficiaries give at least some of the cash benefits to a family carer (Glendinning and Igl 2009). Publicly funded care services do not cover all care needs, but care recipients are expected to make co-payments albeit at a low level (Grages et al. 2021) unless informal carers fill the gap.

### SES Differences in receiving care

#### Theoretical framework

Andersen’s (1995) model of health care use can be applied to predict social care use (Floridi et al. 2021; Suanet et al. 2012). It identifies three factors affecting care use: people’s predispositions to use care, their care needs and the resources enabling access to care. However, this macro-level model does not explain the mechanisms producing these patterns at the individual level. To address this, we complement it with a rational-choice approach to develop individual-level arguments (Kuppler and Wagner 2020). We posit that individuals use formal or informal care if it is available and its subjective cost–benefit relationship is better than for the alternatives. Key costs include financial and emotional burdens, such as overcoming negative attitudes to formal care, which are often present in familistic societies (Schneekloth et al. 2017; Casado et al. 2011). The costs of going without care consist of the resulting difficulties with carrying out activities of daily living.

#### SES differences in the availability of care and attitudes to care

SES differences in care availability arise from structural factors, including purchasing power, the design of the LTCSs, and the availability of children and spouses. Purchasing power is needed to access formal care on the private market. This is important in England because care is means-tested. However, people just above the eligibility threshold for publicly funded care might be reluctant to spend their limited funds on care. People from low-SES groups might pass the means test, but they might not receive formal care because of the restrictive eligibility criteria. The German LTCS does not consider the older person’s financial situation when determining their eligibility for publicly funded care.

Structural factors also shape the availability of informal care. On the one hand, older people in higher SES groups are more likely to live with a partner than those in lower-SES groups.^3^ On the other hand, SES differences in the geographical distance between children and parents (Chan and Ermish 2015; Kalmijn 2006) reduce the availability of informal carers for high-SES groups compared to low-SES groups. In addition, lower opportunity costs make it easier for low-income groups to reduce paid work for caregiving compared to other groups (Saraceno 2010; Sarasa and Billingsley 2008). In Germany, the decision is further supported by the availability of cash benefits for family carers. This differs from England, where Carers Allowance is available only if caregiving becomes the person’s main economic activity.

Filial obligations also contribute to SES differences in receiving informal care (Kalmijn and Saraceno 2008). Lower-educated parents have higher expectations of filial care (Klie and Blinkert 2002), especially in familistic societies (Kalmijn and Saraceno 2008). While Germany used to be regarded as a familistic society (Suanet et al. 2012), these values may have weakened, as evidenced by largely removing children’s financial obligations to support their parents in 2020. Our analysis of the 2017 European Values Survey (EVS 2022) (cf. Online Appendix Tables A1 and A2) shows a slightly stronger sense of filial obligation in Germany compared to Britain, and a negative association between the level of education and the sense of filial obligation in Germany but not in Britain. The weaker support for filial obligations in England aligns with liberal welfare regimes, where people exhibit a stronger orientation to generational autonomy (Kalmijn and Saraceno 2008).

**Table 1:** Overview of hypotheses

	Few care needs	Intensive care needs
Probability of …		
Receiving informal care	**H1**: Low SES grp > high SES grp	**H4**: Low SES grp > high SES grp
Receiving formal care	**H2**: Low SES grp = high SES grp	**H5**: Low SES grp < high SES grp
Having unmet care needs	**H3**: Low SES grp < high SES grp	**H6**: Low SES grp = high SES grp

**Table 2:** Descriptive Statistics (weighted column %)

	All		Has ADL need	
	Germany *N* = 1,610	England *N* = 1,198	Germany *N* = 575	England *N* = 401
Help needed with ADLs				
Activity	Per cent ‘only with (a little) help’	Per cent ‘having difficulty’	Per cent ‘only with (a little) help’	Per cent ‘having difficulty’
Eating (ELSA: ‘…such as cutting up food’)	4.7	6.8	14.3	18.7
Getting in or out of bed	8.7	11.2	26.1	30.6
Getting dressed and undressed (ELSA: ‘…including putting on shoes and socks’)	17.8	24.9	52.0	68.2
Showering or bathing	25.6	19.9	77.4	54.4
Using the toilet (ELSA: ‘…including getting up or down’, NRW80 + : ‘…reaching early enough’)	5.3	9.0	16.1	24.7
Walking^a^ (ELSA: ‘… across a room’)	18.1	9.5	54.6	26.0

	Per cent	Per cent	Per cent	Per cent
Number ADL needs: *^b^ *^d^				
0	67	64		
1–2	21	26	62	71
3–6	13	11	38	29
Age: **^b^ **^d^				
80–84	56	53	44	41
85–89	30	29	36	28
90+	13	18	21	31
Has child: *^b^	87	91	88	89
Gendered living arrangements: *^b^ **^d^				
Male, not alone	28	28	22	28
Male, living alone	11	13	9	14
Female, not alone	20	23	25	22
Female, living alone	41	35	44	36
Education:**^b^ **^d^				
Low	26	46	37	51
Medium	54	43	52	41
High	20	11	11	8
Income: **^b^				
< €1500	50	62	60	63
€1,500–1,999	21	19	20	22
> €1,999	29	19	20	15
Wealth: **^b^ ***^d^				
< €12,500	51	35	64	39
€12,500–24,999	12	13	10	13
€25,000+	38	52	26	48
Home ownership^c^ **^b^ ***^d^	45	75	33	69

Data sources: NRW80+ and wave 8 of ELSA

^a^People who are able to walk ‘with a little help’ are not counted as needing help in Germany

^b^Test of country difference—full sample: ‘*’ *p* < 0.05; ‘**’ *p* < 0.01

^c^13 cases are missing in the full sample and 8 in the sample with ADL needs

^d^Test of country difference—people with ADL need: ‘*’ *p* < 0.05; ‘**’ *p* < 0.01; ‘***’ *p* < 0.001

Empirical research into SES differences in receiving informal care has found that people with a low level of education (Blomgren et al. 2012; Broese van Groenou et al. 2006; Kalmijn and Saraceno 2008; Larsson and Silverstein 2004), low wealth or income (Floridi et al. 2021) or a low social class (Broese van Groenou et al. 2006, for Britain) are more likely to receive informal care than other groups. In Belgium, receiving informal care was unrelated to the educational level (Broese van Groenou et al. 2006).

Patterns in formal care use are more mixed according to past research. In Albertini and Pavolini’s (2017) study, SES differences in receiving formal care differ by care regime. In Germany and Italy ('cash-for-care countries'), income is positively associated with receiving formal care (see also Floridi et al. 2021). In France and Denmark, which are categorized as LTCSs with a more universalistic provision of care services, there is no association between income and receiving formal care. The study by Broese van Groenou et al. (2006) found that in Italy, respondents in the low-SES group had higher odds of receiving formal help than those in the high-SES group, whereas in Great Britain, the Netherlands and Belgium, no association between SES and receiving formal care emerged. In sum, financial resources often correlate with receiving formal care, but the strength of this association depends on individual and country properties. In an earlier study using NRW80+ , Zimmermann (2022) reported no SES differences in using any care by people aged 80+ living in the community. Unmet care needs were observed in low-income groups (Desai et al. 2001), or there was no association between wealth and unmet care needs (Dunatchik et al. 2019; Vlachantoni 2019).

#### SES differences in care use for people with high- and low-intensity care needs

When people assess the costs and benefits of different types of care, they face different options depending on the intensity of their care needs. People with few care needs might consider the option of going without any care. They might regard the costs of coping on their own as lower than accepting formal care, be it for the expense or the dependency on strangers (Casado 2011). People with a high SES might cope better without care than people from low-SES groups because they can afford higher-quality technical aids and home adaptations to facilitate life without receiving care, or they might obtain them more easily. The more care needs people have, the more costly this option gets. With high-intensity care needs, the older person must exploit more fully the available sources of care. High levels of care needs might override the reluctance to rely on formal or informal care.

Drawing together the different arguments, we propose six hypotheses, listed in Table 1. Although the hypotheses apply to both countries, they reflect different country contexts. In England, SES differences in receiving informal care derive exclusively from the geographical distance to children, whereas in Germany, filial obligations and cash benefits also play a role. SES differences in using formal care arise from means testing in England and from compensating for SES differences in receiving informal care in Germany.

## Methods

### Data

We use data from the Survey of ‘Quality of life and well-being of the very old in North Rhine-Westphalia’ (NRW80+) (Hansen et al. 2021) and wave 8 of the English Longitudinal Study of Ageing (ELSA) (Clemens et al. 2019).^4^ NRW80+ collected data for an age-stratified representative sample of individuals aged 80+ living in NRW. The 1,863 interviews took place in 2017/8. ELSA is a panel survey that started in 2002 with a representative sample of the English population aged 50 and over living in private households. We analyse the data collected in 2017 (wave 8), matching the survey year of NRW80+. Early waves of ELSA did not collect sufficiently detailed information about receiving care to enable parallel analyses with NRW80+ . After dropping individuals in residential settings, non-core members of ELSA, individuals younger than 80 years, 34 cases with missing values and 62 people living with ‘Other’ people, the German sample comprises 1,610 individuals and the English sample 1,198 individuals.^5^ We exclude older people who live in communal establishments because these are not included in the ELSA sample. Our analyses are further restricted to individuals who reported a difficulty with an activity of daily living (ADL), because ELSA collected information about receiving help only if the respondent reported a difficulty with an ADL or an instrumental activity of daily living (IADL), and NRW80+ asked only about ‘care’, not ‘help’. This reduced the analysis samples to 575 individuals from Germany and 401 individuals from England. We apply weights to all analyses so that the findings can be generalized to the populations of people aged 80+ living in the community in the respective country.

### Variables

#### Care needs

Both surveys have asked respondents about needing help with selected ADLs. ELSA asked respondents whether they ‘have difficulty doing the activities’ or not. NRW80+ asked respondents whether they were able to do the activities ‘only with help’, ‘with a little help’ or ‘without help’. We categorize people as having a care need if they reported a difficulty (ELSA) or needing (a little) help (NRW80+).^6^ The variable ‘Number of ADL needs’ counts the six ADLs that are included in both surveys (cf. Table 2) and operationalizes the intensity of care needs. We refer to one or two care needs as ‘few’ care needs and three to six care needs as ‘many’ care needs.

#### Receiving care

ELSA respondents identified helpers from lists of formal and informal helpers. In NRW80+, a person received informal care if they received care ‘privately…, for example from a relative or an acquaintance’. The respondent received formal care if they received homecare or attended a day care institution. Because the NRW80+ questions refer to ‘care’ (‘Pflege’), not the broader concept of ‘help’, we restrict care receipt in ELSA to help provided with mobility, washing or eating and ignore helpers who only provided help with IADLs. The indicators for ‘Informal care’ and ‘Formal care’ take value one if the respondent received the respective type of care and zero otherwise.

Our construction of the variable ‘unmet (care) need’ follows common practice (Calderon-Jaramillo and Zueras 2023; Casado et al. 2011; Maplethorpe et al. 2015; Vlachantoni 2019). It takes value one if the respondent reported at least one ADL need but did not receive any care, and value zero otherwise.

**Fig. 1:**
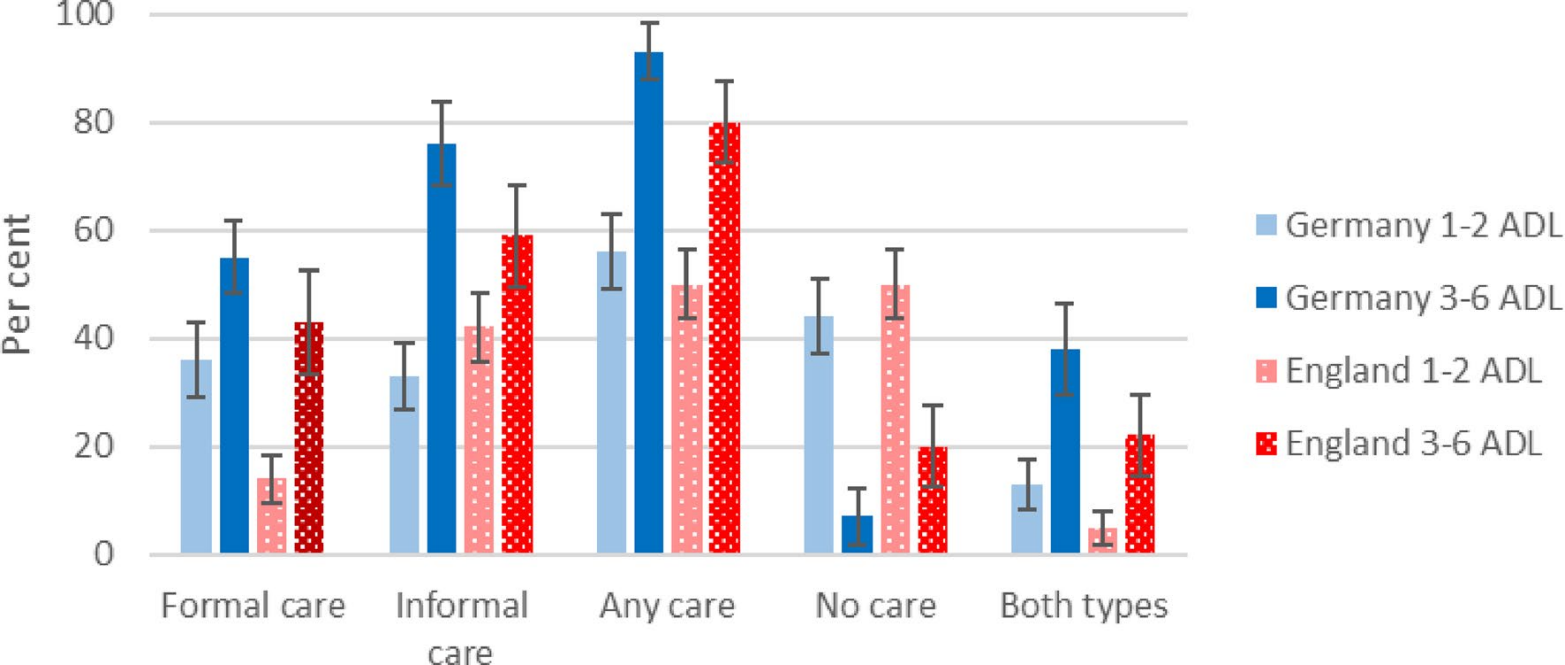
Per cent of individuals with at least one ADL need receiving care, by country, type of care and number of care needs: percentages and 95% confidence intervals

Figure 1 gives the proportions of older people who received different types of care. Slightly more than half of people with few care needs received any care. In England, this is overwhelmingly informal care, whereas in Germany about equal proportions of people received formal and informal care. Higher proportions of people with many care needs received care—93% in Germany and 80% in England. Only minorities of people with many care needs received both formal and informal care—38% in Germany compared to 22% in England.

#### Independent variables

We distinguish between people who lived alone and those who lived with a partner and/or a child. By interacting the indicator with gender, we create a four-category variable, with ‘Male, not living alone’ as reference category.

The dummy variable ‘Has child’ takes value one if the respondent has a living child, and value zero otherwise. The age variable has three categories: 80–84 years old, 85–89 years old, and 90 years or older. We consider three dimensions of SES. Both surveys provide a three-category classification of educational attainment based on the ISCED classification (OECD 2015). Both surveys collected the equivalised net income and the net non-housing wealth of the household (NRW80+) or benefit-unit (ELSA).^7^ We aggregated these into three-category variables for income and wealth, respectively. The categories of the wealth variable match as closely as possible the criteria for means testing in England. Finally, we include a dummy variable for home ownership. Table 2 gives the distributions of the variables for the whole samples and the analysis samples of people aged 80+ with at least one care need.

#### Analysis strategy

We estimate logit models for receiving informal care, formal care and having unmet care needs for people with at least one ADL need. Table 3 gives estimates for the full model with the level of education as a covariate. The Online Appendix gives stepwise models (Tables A3 to A8) as well as the corresponding tables for income and wealth (Tables A9 and A10). It also presents models with an interaction between the number of care needs and the different SES indicators, testing our conjecture about the intensity of care needs as a moderator of SES differences (Tables A11 to A13). All models are estimated separately for the two countries.

**Table 3:** Logit models for receiving informal care, formal care or having unmet care needs^a^

	Informal care				Formal care				Unmet needs			
	Germany		England		Germany		England		Germany		England	
	Coeff	*t*	Coeff	*t*	Coeff	*t*	Coeff	*t*	Coeff	*t*	Coeff	*t*
N of needs (ref = 1)												
2	1.029^***^	*3.69*	0.925^**^	*3.02*	0.892^**^	*3.23*	0.823^*^	*1.99*	− 1.137^***^	*− 3.87*	− 0.927^**^	*− 3.08*
3	1.938^***^	*5.05*	1.205^**^	*2.98*	0.963^**^	*3.05*	0.619	*1.19*	− 2.396^***^	*− 4.14*	− 0.999^**^	*− 2.63*
4	1.915^***^	*3.98*	1.529^***^	*3.36*	1.910^***^	*4.63*	1.725^***^	*3.63*	− 2.398^***^	*− 4.04*	− 2.422^***^	*− 4.83*
5	2.906^***^	*5.09*	0.945	*1.62*	1.772^***^	*3.53*	2.341^***^	*3.58*	− 3.970^***^	*− 4.31*	− 2.209^***^	*− 4.01*
6	4.657^***^	*4.58*	0.939^†^	*1.73*	1.393^**^	*2.77*	3.524^***^	*6.22*				
Age (ref = 80–84)												
85–89	0.442	*1.44*	0.201	*0.69*	0.105	*0.40*	− 0.281	*− 0.72*	− 0.708^*^	*− 2.38*	− 0.289	*− 0.98*
90+	0.621^*^	*2.09*	0.800^*^	*2.53*	− 0.105	*− 0.39*	0.578	*1.55*	− 0.833^**^	*− 2.61*	− 1.110^***^	*− 3.36*
Male, not alone (ref)												
Male, lives alone	− 0.066	*− 0.17*	− 1.819^***^	*− 4.41*	0.917^*^	*2.40*	0.786^†^	*1.75*	− 0.777^*^	*− 1.99*	1.722^***^	*4.22*
Female, not alone	− 0.297	*− 1.03*	− 0.128	*− 0.39*	0.214	*0.63*	0.162	*0.26*	0.054	*− 0.17*	0.058	*0.17*
Female, lives alone	− 0.768^**^	*− 2.67*	− 1.548^***^	*− 4.50*	1.236^***^	*4.31*	1.617^***^	*3.82*	− 0.345	*− 1.01*	0.955^**^	*2.73*
Has a child	0.457	*1.41*	0.596	*1.43*	− 0.118	*− 0.40*	− 0.871^*^	*− 2.16*	− 0.355	*− 1.11*	0.188	*0.50*
Education (ref: Low)												
Middle	− 0.363	*− 1.19*	− 0.159	*− 0.62*	0.166	*0.72*	0.011	*0.03*	0.277	*0.88*	0.194	*0.73*
High	− 0.676^*^	*− 2.10*	− 0.887^*^	*− 2.17*	-0.133	*-0.42*	0.280	*0.56*	0.799^*^	*2.33*	0.884^*^	*2.13*
Owns home	-0.252	*-1.13*	-0.087	*-0.32*	-0.103	*-0.47*	-0.230	*-0.72*	0.295	*1.08*	0.333	*1.23*
Constant	− 1.025^*^	*− 2.23*	− 0.748	*− 1.02*	− 1.705^***^	*− 3.80*	− 2.259^***^	*− 3.88*	0.792^†^	*1.65*	− 0.423	*-0.81*
*N*	573		395		573		395		573		395	
*Dependent var. ‘yes’*	50%		47%		43%		23%		30%		41%	

^a^Figure 2, Panel A, shows selected corresponding predicted margins

^†^*p* < 0.10, ^*^p < 0.05, ^**^p < 0.01, ^***^p < 0.001

## Findings

**Fig. 2:**
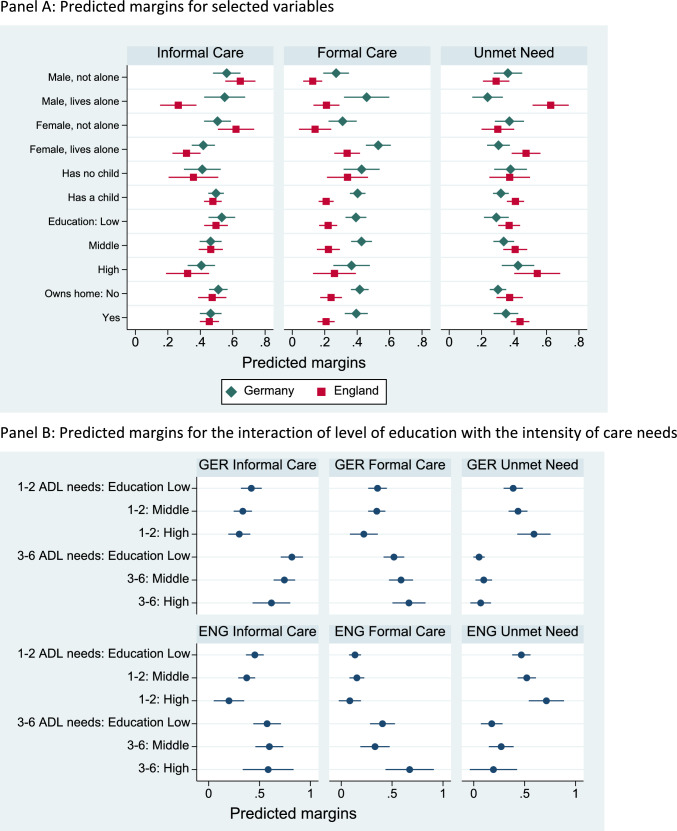
Predicted margins of receiving informal care, formal care or having an unmet need: models with level of education. Note: Country-specific models, controlling for age, number of ADL needs, gender and living arrangements, having a child, level of education, home ownership. Full model estimates are shown in Table 3 and Table A11 in the Online Appendix. Table A14 of the Online Appendix reports significance tests for SES differences in Panel B

Table 3 gives the estimated effects of the level of education on the three outcome variables. The marginal probabilities associated with selected variables are shown in Fig. 2, Panel A. In both countries, the likelihood of receiving informal care declines with the level of education. There is no educational gradient in receiving formal care in either country. In both countries, people with a high level of education are more likely to have unmet care needs compared to people with a low level of education, in contrast to Vlachantoni’s (2019) findings for England. Homeownership did not make any difference to receiving care in the two countries, in contrast to previous research (Albertini and Pavolini 2017; Broese van Groenou et al. 2006).

**Fig. 3:**
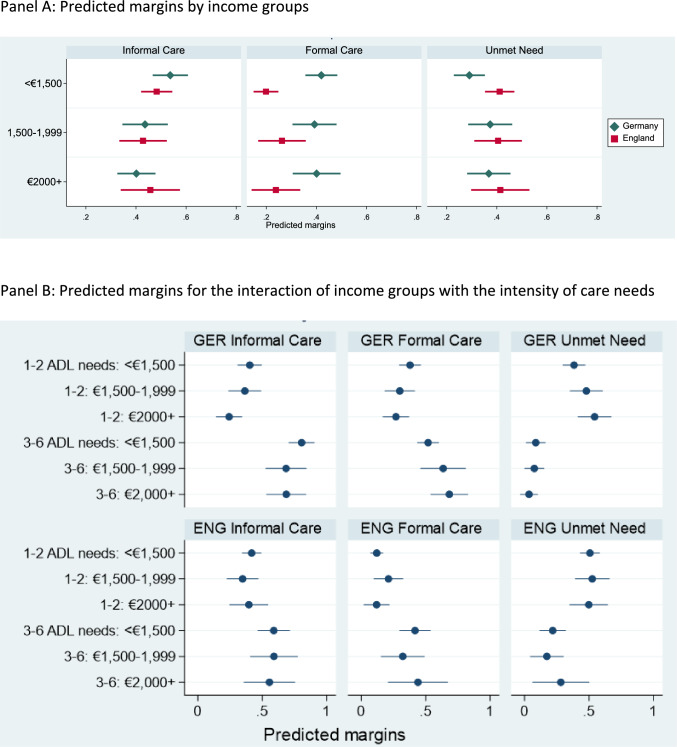
Predicted margins of receiving informal care, formal care or having unmet needs: models with income groups. Note: Country-specific models, controlling for age, number of ADL needs, gender and living arrangements, having a child, income. Full model estimates in Appendix Tables A9 and A12. Table A14 of the Online Appendix gives significance tests for SES differences in Panel B

**Fig. 4:**
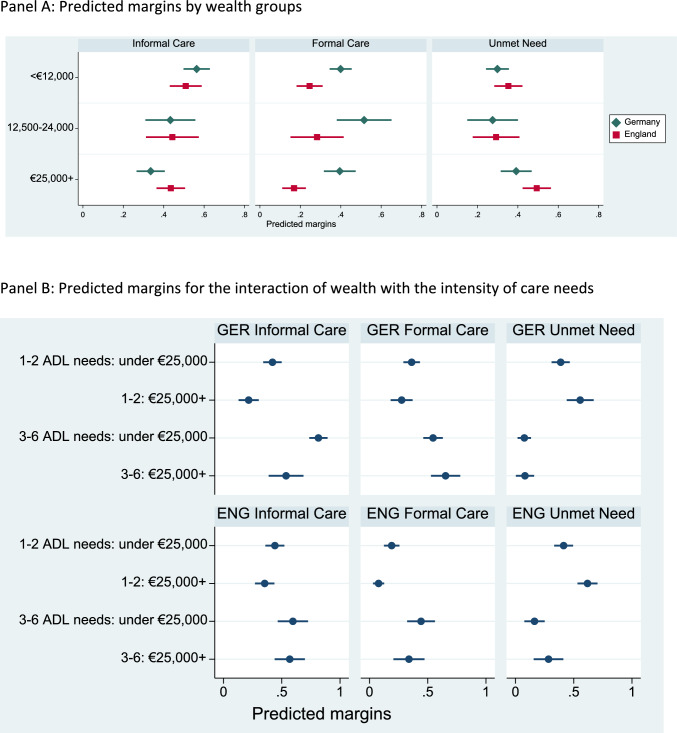
Predicted margins of receiving informal care, formal care or having unmet needs: models with wealth groups. Note: Country-specific models, controlling for age, number of ADL needs, gender and living arrangements, having a child, wealth. Full model estimates in Appendix Tables A10 and A13. Table A14 of the Online Appendix gives significance tests for SES differences in Panel B

The upper panel of Fig. 3 gives the predicted margins associated with income groups. These are estimated from the models in Table 3 with indicators for income replacing those for education. For Germany, they broadly replicate the patterns observed for education groups. In England, there is no significant association between income and receiving care or having unmet needs. The upper panel of Fig. 4 gives the corresponding estimates when using wealth as SES indicator. Again, the German analyses broadly replicate the earlier patterns. In England, wealth is not associated with receiving informal care, but the wealthiest people are more likely to have unmet needs than people in the lowest wealth group.

The findings about a negative education gradient in receiving informal care support earlier research. In the models with income and wealth, SES differences are only significant in Germany. The analyses show no statistically significant effect of any SES indicator on receiving formal care. In particular, they do not show the positive association between income and receiving formal care reported by Albertini and Pavolini (2017) for Germany, but we analyse an older population and only people with care needs.

Panel B of Fig. 2 displays the marginal probabilities derived from models with an interaction effect between the number of care needs and the level of education. For people with few care needs, we see a negative educational gradient of receiving informal care, but only in England is the difference between high- and low-SES groups statistically significant at the 5% level. This is partly in line with **H1**. **H2** is supported because there are no significant differences in receiving formal care by the level of education. The likelihood for unmet care needs is higher for highly educated people than for people with a low level of education, supporting **H3**.

For people with many care needs, some different patterns emerge. In Germany, the likelihood of receiving informal care is higher for people with a low level of education than those with a high level of education, which is in line with **H4.** In England, there is no educational gradient in using informal care, in contrast to **H4**. The likelihood of using formal care is higher among highly educated people than among low educated people in both countries, which aligns with **H5**. **H6** is also supported: there is no difference in the likelihood of unmet needs between educational groups in both countries.

The lower parts of Figs. 3 and 4 give the corresponding estimates for different income and wealth groups. The lower part of Table 4 summarizes the results from significance tests relating to the six hypotheses.

**Table 4:** Overview of hypotheses and hypothesis tests

	Few care needs	Many care needs
Hypotheses: Probability of …		
Receiving informal c	**H1**: Low SES grp > high SES grp	**H4**: Low SES grp > high SES grp
Receiving formal care	**H2**: Low SES grp = high SES grp	**H5**: Low SES grp < high SES grp
Having unmet needs	**H3**: Low SES grp < high SES grp	**H6**: Low SES grp = high SES grp
Findings at 5% level of significance		

	Education	Income	Wealth	Education	Income	Wealth
Germany						
Receiving informal c	✗	✓	✓	✓	✗	✓
Receiving formal care	✓	✓	✓	✓	✓	✗
Having unmet needs	✓	✓	✓	✓	✓	✓
England						
Receiving informal c	✓	✗	✗	✗	✗	✗
Receiving formal care	✓	✓	✗	✓	✗	✗
Having unmet needs	✓	✗	✓	✓	✓	✓

‘✗’ not supported, ‘✓’ supported

Taken together, there is support for an SES gradient in using informal care in Germany irrespective of care intensity (**H1** and **H4**). In England, there is some support for such a gradient when care needs are low (**H1**), but there is no support when care needs are high (**H4**). The hypothesis of no SES difference in the likelihood of receiving formal care for people with few care needs (**H2**) is mostly supported. This suggests that two trends balance each other out—a higher likelihood of not receiving informal care in high-SES groups and a better ability to cope without care in these groups. In England, there is only partial support for **H5**—that there are SES differences in receiving formal care when care needs are high. The hypothesis relied on the assumption that high-SES groups can purchase the care they need on the market, leading to a higher likelihood of using formal care than people with less income or wealth, including people relying on publicly paid care. This assumption is not supported by the analyses. The hypotheses about unmet care needs (**H3** and **H6**) are mostly supported in both countries.

## Discussion and conclusion

The growing number of people aged 80 + living in the community has raised concerns about the adequacy of their care and social inequalities in their care use. We propose that SES patterns in care use change with the intensity of care needs, as different levels of frailty offer different options regarding their care. We have examined the proposition for Germany and England.

Among people with few care needs, we observe a consistent association between SES and care use in both countries, despite differences in the LTCSs. In particular, people from lower SES groups are more likely to receive informal care, whereas those from high-SES groups are most likely to experience unmet care needs. This pattern holds across several SES indicators. Previous research has reported similar findings (Broese van Groenou et al. 2006; Hoogendijk et al. 2014; Kalmijn and Saraceno 2008). Importantly, we show that they only apply to people with few care needs.

For older people with many care needs, we find country-specific SES patterns that we link to cultural norms about filial obligations and different LTCSs. Germany’s LTCS combines elements of familialism and de-familialization, allowing cash benefits to be given to informal carers, used to purchase formal care services, or both (Verbakel et al. 2023; Saraceno 2010). These flexible arrangements appear to produce a negative SES gradient in using informal care, possibly reflecting stronger filial norms (Kalmijn and Saraceno 2008) and incentives from cash benefits (Unger et al. 2015). The term ‘incentive’ arguably mischaracterizes the reality of some low-income families who lack feasible alternatives due to the limits of the available formal care (Saraceno 2010). For them, cash benefits serve to mitigate income losses associated with reduced hours of paid work due to caregiving responsibilities.

In England, we find hardly any significant SES differences in care use for people with many care needs. This likely reflects little choice regarding their care and no SES differences in views about filial obligations. The moderate margins of having unmet care needs in all SES groups suggest some success of the English LTCS in targeting publicly paid services at people with the greatest need. However, a small number of people with many care needs receive no care—mostly people who live alone (analyses not shown). 

We observe an increased use of formal care in the highest educational group but not for people with high income or wealth in England. The latter findings diverge from our hypothesis. It could indicate practical difficulties of accessing the care market, preferences to retain assets in the family or a reluctance to spend limited funds. The underlying causes of this restrained care purchasing behaviour by relatively well-off people in England merit further investigation. 

Our study has several limitations. Using two different surveys risks inconsistencies in the main measures. Rutherford and Bu (2018) have demonstrated the importance of framing and question wording for measuring informal care. Although we have exploited the considerable detail of ELSA to tailor the variables to those in NRW80+ , discrepancies might remain. In addition, our binary measures of care receipt do not capture the hours of care nor its adequacy. Our frailty measures are subjective rather than clinically assessed. Furthermore, the study focuses on help with ADLs, neglecting help with IADLs, which can become increasingly challenging in this age group.

Despite these limitations, our study extends existing research by identifying a common SES pattern of care use among people with few care needs, independent of country context. It is remarkable that we found no SES differences in unmet needs among those with high care needs, despite differing LTCSs. SES disparities are stronger in Germany than in England. Although such differences may reflect personal choice, they can lead to further social inequalities, for example, regarding informal carers’ health and public resource allocation. 

## Supplementary Information

Below is the link to the electronic supplementary material.

Supplementary file1 (DOCX 100 KB)

## Data Availability

The English Longitudinal Study of Ageing has been accessed through the UK Data Service. It has been collected by a team of researchers at University College London: Banks, J., Batty, G. David, Breedvelt, J., Coughlin, K., Crawford, R., Marmot, M., Nazroo, J., Oldfield, Z., Steel, N., Steptoe, A., Wood, M., Zaninotto, P. (2024). English Longitudinal Study of Ageing: Waves 0–10, 1998–2023. [data collection]. 43rd Edition. UK Data Service. SN: 5050, DOI: http://doi.org/10.5255/UKDA-SN-5050-30 . The Survey of Quality of Life and Well-Being of the Very Old in North Rhine-Westphalia has been collected by a team of researchers at Cologne University, including one of the authors. It is accessible through GESIS (Leibniz Institute for the Social Sciences): GESIS, Cologne. ZA7558 Data file Version 2.0.0, https://doi.org/10.4232/1.13978.

## Footnotes

1 The amount does not include the value of the person’s property if they live in the property.

2 From analysing wave 8 of Understanding Society (University of Essex, 2024), we estimate that about 6% of informal carers for a spouse aged 80+ or a parent or parent-in-law aged 80+ receive Carers Allowance. Carer’s allowance amounted to £67.60/w (about €76/w) at the time of our surveys. Since then, it has increased to £81.90/w.

3 The association is significant in our data sets.

4 We did not use the German SHARE data because it includes too few people aged 80+.

5 Individuals who lived with ‘Other’ people are a heterogeneous group and too few for a separate analysis.

6 We made one exception to this rule and excluded the large number of people who needed ‘a little’ help with walking, ‘for example a walking stick’.

7 We imputed missing values for wealth and income in NRW80+. ELSA data for wealth and income include imputations by the data providers. We transformed British Pounds into Euros, applying the exchange rate on 15.11.2017: £100 = €111.22.
