# Supplementary material for: Socio-economic differences in receiving care by the over-80s in Germany and England: intensity of care needs as a moderator
Source: Eur J Ageing. 2025 Jun 13;22(1):27. doi: 10.1007/s10433-025-00864-y (PMC12165915; doi:10.1007/s10433-025-00864-y)
Supplement: Supplementary file 1 — Supplementary file1 (DOCX 100 KB) [file 10433_2025_864_MOESM1_ESM.docx]

**Online Appendix**

Article title: Socio-economic Differences in Receiving Care by the Over-80s in Germany and England: Intensity of care need as a moderator

Journal: European Journal of Ageing

Table A1: Attitudes to children’s duty to provide long-term care for parents (Percent)

|  | Germany | Great Britain |
| --- | --- | --- |
| Agree strongly | 9 | 6 |
| Agree | 36 | 34 |
| Neither | 19 | 20 |
| Disagree | 28 | 34 |
| Disagree strongly | 8 | 6 |
|  |  |  |
| Total | 100 | 100 |

Data: European Values Survey 2017

Notes: N= 2,127 (Germany), N=1,784 (Great Britain)

Chi^2^ (4) = 55.8, p<0.000

Table A2: Attitudes to children’s duty to provide long-term care for parents, by country and level of education (Percent)

|  | Germany | | | Great Britain | | |
| --- | --- | --- | --- | --- | --- | --- |
|  | Lower | Middle | Upper | Lower | Middle | Upper |
| Agree strongly | 19 | 7 | 9 | 7 | 7 | 5 |
| Agree | 34 | 37 | 37 | 30 | 27 | 29 |
| Neither | 16 | 17 | 18 | 21 | 21 | 23 |
| Disagree | 23 | 29 | 29 | 35 | 35 | 34 |
| Disagree strongly | 8 | 9 | 6 | 7 | 10 | 9 |
|  |  |  |  |  |  |  |
| Total | 100 | 100 | 100 | 100 | 100 | 100 |
|  | (N=420) | (N=1912) | (N=1290) | (N=1367) | (N=723) | (N=2103) |
| Chi2 (8) | p<0.000 | | | p=0.38 | | |

Data: European Values Survey 2017 and World Values Survey wave 7 (2017-2022)

Sample: N=3,622 (Germany), N=4,193 (Great Britain)

Table A3: Informal care in Germany: stepwise models

|  | (1) |  | (2) |  | (3) |  | (4) |  | (5) |  |
| --- | --- | --- | --- | --- | --- | --- | --- | --- | --- | --- |
|  | Coef. | t | Coef. | t | Coef. | t | Coef. | t | Coef. | t |
| ADL needs: 1 | 0 |  | 0 |  | 0 |  | 0 |  | 0 |  |
| 2 | 1.015^***^ | (3.76) | 1.029^***^ | (3.69) | 1.017^***^ | (3.71) | 1.003^***^ | (3.67) | 1.043^***^ | (3.87) |
| 3 | 1.901^***^ | (5.36) | 1.938^***^ | (5.05) | 1.834^***^ | (5.04) | 1.793^***^ | (4.98) | 1.953^***^ | (5.20) |
| 4 | 1.919^***^ | (4.12) | 1.915^***^ | (3.98) | 1.954^***^ | (4.34) | 1.936^***^ | (4.00) | 1.946^***^ | (4.08) |
| 5 | 2.936^***^ | (5.11) | 2.906^***^ | (5.09) | 2.954^***^ | (4.99) | 2.908^***^ | (5.18) | 2.940^***^ | (4.83) |
| 6 | 4.656^***^ | (4.70) | 4.657^***^ | (4.58) | 4.751^***^ | (4.87) | 4.553^***^ | (4.37) | 4.676^***^ | (4.61) |
| Age: 80-84 | 0 |  | 0 |  | 0 |  | 0 |  | 0 |  |
| 85-89 | 0.448 | (1.45) | 0.442 | (1.44) | 0.423 | (1.37) | 0.338 | (1.11) | 0.442 | (1.44) |
| 90+ | 0.564^+^ | (1.91) | 0.621^*^ | (2.09) | 0.626^*^ | (2.13) | 0.632^*^ | (2.20) | 0.619^*^ | (2.09) |
| Male, not alone | 0 |  | 0 |  | 0 |  | 0 |  | 0 |  |
| Male, lives alone | -0.070 | (-0.17) | -0.066 | (-0.17) | 0.031 | (0.08) | -0.214 | (-0.55) | -0.050 | (-0.12) |
| Female, not alone | -0.138 | (-0.50) | -0.297 | (-1.03) | -0.291 | (-1.03) | -0.191 | (-0.64) | -0.290 | (-0.98) |
| Female, lives alone | -0.573^*^ | (-2.12) | -0.768^**^ | (-2.67) | -0.605^*^ | (-2.27) | -0.718^**^ | (-2.67) | -0.744^*^ | (-2.38) |
| Has a child | 0.486 | (1.42) | 0.457 | (1.41) | 0.482 | (1.46) | 0.351 | (1.02) | 0.454 | (1.39) |
| Education: Low |  |  | 0 |  |  |  |  |  | 0 |  |
| Middle |  |  | -0.363 | (-1.19) |  |  |  |  | -0.360 | (-1.17) |
| High |  |  | -0.676^*^ | (-2.10) |  |  |  |  | -0.678^*^ | (-2.13) |
| Homeowner |  |  | -0.252 | (-1.13) |  |  |  |  | -0.252 | (-1.13) |
| Income: <€1,500 |  |  |  |  | 0 |  |  |  |  |  |
| €1,500-1,999 |  |  |  |  | -0.529 | (-1.64) |  |  |  |  |
| €2,000+ |  |  |  |  | -0.720^*^ | (-2.46) |  |  |  |  |
| Wealth: <€12,500 |  |  |  |  |  |  | 0 |  |  |  |
| €12,500-24,999 |  |  |  |  |  |  | -0.679^+^ | (-1.78) |  |  |
| €25,000+ |  |  |  |  |  |  | -1.234^***^ | (-4.82) |  |  |
| Uses formal care |  |  |  |  |  |  |  |  | -0.076 | (-0.28) |
| Constant | -1.511^***^ | (-3.44) | -1.025^*^ | (-2.23) | -1.217^**^ | (-2.63) | -0.870^+^ | (-1.82) | -1.016^*^ | (-2.19) |
| *N* | 573 |  |  |  |  |  |  |  |  |  |

Table A4: Informal care in England: stepwise models

|  | (1) |  | (2) |  | (3) |  | (4) |  | (5) |  |
| --- | --- | --- | --- | --- | --- | --- | --- | --- | --- | --- |
|  | Coef. | t | Coef. | t | Coef. | t | Coef. | t | Coef. | t |
|  |  |  |  |  |  |  |  |  |  |  |
| ADL needs: 1 | 0 |  | 0 |  | 0 |  | 0 |  | 0 |  |
| 2 | 0.925^**^ | (3.06) | 0.925^**^ | (3.02) | 0.931^**^ | (3.07) | 0.888^**^ | (2.94) | 0.937^**^ | (3.03) |
| 3 | 1.218^**^ | (3.00) | 1.205^**^ | (2.98) | 1.250^**^ | (3.06) | 1.232^**^ | (3.02) | 1.213^**^ | (2.98) |
| 4 | 1.511^***^ | (3.36) | 1.529^***^ | (3.36) | 1.519^***^ | (3.34) | 1.519^***^ | (3.37) | 1.565^***^ | (3.47) |
| 5 | 0.976^+^ | (1.71) | 0.945 | (1.62) | 1.017^+^ | (1.81) | 0.964^+^ | (1.77) | 1.001 | (1.65) |
| 6 | 0.920^+^ | (1.74) | 0.939^+^ | (1.73) | 0.880^+^ | (1.66) | 0.929^+^ | (1.73) | 1.022^+^ | (1.78) |
| Age: 80-84 | 0 |  | 0 |  | 0 |  | 0 |  | 0 |  |
| 85-89 | 0.188 | (0.65) | 0.201 | (0.69) | 0.177 | (0.61) | 0.199 | (0.69) | 0.197 | (0.67) |
| 90+ | 0.854^**^ | (2.69) | 0.800^*^ | (2.53) | 0.845^**^ | (2.64) | 0.904^**^ | (2.79) | 0.813^*^ | (2.57) |
| Male, not alone | 0 |  | 0 |  | 0 |  | 0 |  | 0 |  |
| Male, lives alone | -1.689^***^ | (-4.24) | -1.819^***^ | (-4.41) | -1.676^***^ | (-4.22) | -1.744^***^ | (-4.36) | -1.808^***^ | (-4.37) |
| Female, not alone | 0.006 | (0.02) | -0.128 | (-0.39) | -0.006 | (-0.02) | -0.030 | (-0.09) | -0.124 | (-0.38) |
| Female, lives alone | -1.401^***^ | (-4.37) | -1.548^***^ | (-4.50) | -1.420^***^ | (-4.44) | -1.496^***^ | (-4.37) | -1.522^***^ | (-4.37) |
| Has a child | 0.615 | (1.48) | 0.596 | (1.43) | 0.611 | (1.47) | 0.564 | (1.37) | 0.571 | (1.35) |
| Education: Low |  |  | 0 |  |  |  |  |  | 0 |  |
| Middle |  |  | -0.159 | (-0.62) |  |  |  |  | -0.159 | (-0.62) |
| High |  |  | -0.887^*^ | (-2.17) |  |  |  |  | -0.882^*^ | (-2.16) |
| Homeowner |  |  | -0.087 | (-0.32) |  |  |  |  | -0.092 | (-0.34) |
| Income: <€1,500 |  |  |  |  | 0 |  |  |  |  |  |
| €1,500-1,999 |  |  |  |  | -0.266 | (-0.94) |  |  |  |  |
| €2,000+ |  |  |  |  | -0.124 | (-0.38) |  |  |  |  |
| Wealth: <€12,500 |  |  |  |  |  |  | 0 |  |  |  |
| €12,500-24,999 |  |  |  |  |  |  | -0.325 | (-0.84) |  |  |
| €25,000+ |  |  |  |  |  |  | -0.361 | (-1.31) |  |  |
| Uses formal care |  |  |  |  |  |  |  |  | -0.139 | (-0.40) |
| Constant | -0.860^+^ | (-1.92) | -0.536 | (-1.02) | -0.771^+^ | (-1.65) | -0.564 | (-1.14) | -0.508 | (-0.95) |
| *N* | 395 |  |  |  |  |  |  |  |  |  |

Table A5: Formal care in Germany: stepwise models

|  | (1) |  | (2) |  | (3) |  | (4) |  | (5) |  |
| --- | --- | --- | --- | --- | --- | --- | --- | --- | --- | --- |
|  | Coef. | t | Coef. | t | Coef. | t | Coef. | t | Coef. | t |
|  |  |  |  |  |  |  |  |  |  |  |
| ADL needs: 1 | 0 |  | 0 |  | 0 |  | 0 |  | 0 |  |
| 2 | 0.904^**^ | (3.28) | 0.892^**^ | (3.23) | 0.911^**^ | (3.30) | 0.883^**^ | (3.20) | 0.906^**^ | (3.25) |
| 3 | 0.986^**^ | (3.13) | 0.963^**^ | (3.05) | 0.976^**^ | (3.01) | 0.973^**^ | (3.08) | 0.991^**^ | (2.77) |
| 4 | 1.892^***^ | (4.60) | 1.910^***^ | (4.63) | 1.897^***^ | (4.66) | 1.932^***^ | (4.68) | 1.938^***^ | (4.57) |
| 5 | 1.740^***^ | (3.48) | 1.772^***^ | (3.53) | 1.740^***^ | (3.42) | 1.769^***^ | (3.47) | 1.810^***^ | (3.41) |
| 6 | 1.397^**^ | (2.81) | 1.393^**^ | (2.77) | 1.413^**^ | (2.86) | 1.395^**^ | (2.82) | 1.436^**^ | (2.78) |
| Age: 80-84 | 0 |  | 0 |  | 0 |  | 0 |  | 0 |  |
| 85-89 | 0.094 | (0.35) | 0.105 | (0.40) | 0.086 | (0.33) | 0.103 | (0.39) | 0.110 | (0.41) |
| 90+ | -0.130 | (-0.48) | -0.105 | (-0.39) | -0.129 | (-0.48) | -0.158 | (-0.59) | -0.099 | (-0.36) |
| Male, not alone | 0 |  | 0 |  | 0 |  | 0 |  | 0 |  |
| Male, lives alone | 0.908^*^ | (2.36) | 0.917^*^ | (2.40) | 0.917^*^ | (2.41) | 0.948^*^ | (2.44) | 0.916^*^ | (2.40) |
| Female, not alone | 0.213 | (0.65) | 0.214 | (0.63) | 0.189 | (0.55) | 0.261 | (0.81) | 0.210 | (0.61) |
| Female, lives alone | 1.247^***^ | (4.64) | 1.236^***^ | (4.31) | 1.246^***^ | (4.57) | 1.288^***^ | (4.87) | 1.226^***^ | (4.17) |
| Has a child | -0.139 | (-0.47) | -0.118 | (-0.40) | -0.138 | (-0.48) | -0.181 | (-0.60) | -0.112 | (-0.37) |
| Education: Low |  |  | 0 |  |  |  |  |  | 0 |  |
| Middle |  |  | 0.166 | (0.72) |  |  |  |  | 0.161 | (0.69) |
| High |  |  | -0.133 | (-0.42) |  |  |  |  | -0.141 | (-0.44) |
| Homeowner |  |  | -0.103 | (-0.47) |  |  |  |  | -0.106 | (-0.49) |
| Income: <€1,500 |  |  |  |  | 0 |  |  |  |  |  |
| €1,500-1,999 |  |  |  |  | -0.131 | (-0.47) |  |  |  |  |
| €2,000+ |  |  |  |  | -0.091 | (-0.33) |  |  |  |  |
| Wealth: <€12,500 |  |  |  |  |  |  | 0 |  |  |  |
| €12,500-24,999 |  |  |  |  |  |  | 0.550 | (1.55) |  |  |
| €25,000+ |  |  |  |  |  |  | -0.021 | (-0.10) |  |  |
| Uses informal care |  |  |  |  |  |  |  |  | -0.068 | (-0.26) |
| Constant | -1.647^***^ | (-4.31) | -1.705^***^ | (-3.80) | -1.597^***^ | (-4.01) | -1.690^***^ | (-4.18) | -1.685^***^ | (-3.80) |
| *N* | 573 |  |  |  |  |  |  |  |  |  |

Table A6: Formal care in England: stepwise models

|  | (1) |  | (2) |  | (3) |  | (4) |  | (5) |  |
| --- | --- | --- | --- | --- | --- | --- | --- | --- | --- | --- |
|  | Coef. | t | Coef. | t | Coef. | t | Coef. | t | Coef. | T |
|  |  |  |  |  |  |  |  |  |  |  |
| ADL needs: 1 | 0 |  | 0 |  | 0 |  | 0 |  | 0 |  |
| 2 | 0.833^*^ | (2.03) | 0.823^*^ | (1.99) | 0.799^+^ | (1.94) | 0.746^+^ | (1.78) | 0.832^*^ | (2.01) |
| 3 | 0.629 | (1.21) | 0.619 | (1.19) | 0.579 | (1.10) | 0.651 | (1.23) | 0.634 | (1.22) |
| 4 | 1.764^***^ | (3.64) | 1.725^***^ | (3.63) | 1.751^***^ | (3.70) | 1.673^***^ | (3.34) | 1.743^***^ | (3.64) |
| 5 | 2.344^***^ | (3.58) | 2.341^***^ | (3.58) | 2.269^***^ | (3.38) | 2.313^***^ | (3.56) | 2.354^***^ | (3.55) |
| 6 | 3.562^***^ | (6.23) | 3.524^***^ | (6.22) | 3.664^***^ | (6.29) | 3.452^***^ | (6.07) | 3.536^***^ | (6.30) |
| Age: 80-84 | 0 |  | 0 |  | 0 |  | 0 |  | 0 |  |
| 85-89 | -0.298 | (-0.77) | -0.281 | (-0.72) | -0.298 | (-0.77) | -0.344 | (-0.89) | -0.279 | (-0.72) |
| 90+ | 0.530 | (1.44) | 0.578 | (1.55) | 0.539 | (1.45) | 0.549 | (1.45) | 0.587 | (1.55) |
| Male, not alone | 0 |  | 0 |  | 0 |  | 0 |  | 0 |  |
| Male, lives alone | 0.825^+^ | (1.85) | 0.786^+^ | (1.75) | 0.810^+^ | (1.80) | 0.786^+^ | (1.80) | 0.762 | (1.63) |
| Female, not alone | 0.198 | (0.33) | 0.162 | (0.26) | 0.253 | (0.41) | 0.080 | (0.13) | 0.161 | (0.26) |
| Female, lives alone | 1.655^***^ | (3.98) | 1.617^***^ | (3.82) | 1.703^***^ | (4.00) | 1.499^***^ | (3.59) | 1.595^***^ | (3.59) |
| Has a child | -0.871^*^ | (-2.11) | -0.871^*^ | (-2.16) | -0.855^*^ | (-2.07) | -0.962^*^ | (-2.29) | -0.862^*^ | (-2.13) |
| Education: Low |  |  | 0 |  |  |  |  |  | 0 |  |
| Middle |  |  | 0.011 | (0.03) |  |  |  |  | 0.005 | (0.02) |
| High |  |  | 0.280 | (0.56) |  |  |  |  | 0.269 | (0.53) |
| Homeowner |  |  | -0.230 | (-0.72) |  |  |  |  | -0.232 | (-0.73) |
| Income: <€1,500 |  |  |  |  | 0 |  |  |  |  |  |
| €1,500-1,999 |  |  |  |  | 0.474 | (1.22) |  |  |  |  |
| €2,000+ |  |  |  |  | 0.304 | (0.75) |  |  |  |  |
| Wealth: <€12,500 |  |  |  |  |  |  | 0 |  |  |  |
| €12,500-24,999 |  |  |  |  |  |  | 0.248 | (0.50) |  |  |
| €25,000+ |  |  |  |  |  |  | -0.608^+^ | (-1.71) |  |  |
| Uses informal care |  |  |  |  |  |  |  |  | -0.066 | (-0.19) |
| Constant | -2.503^***^ | (-4.76) | -2.359^***^ | (-3.88) | -2.690^***^ | (-4.96) | -2.065^***^ | (-3.41) | -2.330^***^ | (-3.70) |
| *N* | 395 |  |  |  |  |  |  |  |  |  |

Table A7: Care deficit in Germany: stepwise models

|  | (1) |  | (2) |  | (3) |  | (4) |  |
| --- | --- | --- | --- | --- | --- | --- | --- | --- |
|  | Coef. | t | Coef. | t | Coef. | t | Coef. | t |
|  |  |  |  |  |  |  |  |  |
| ADL needs: 1 | 0 |  | 0 |  | 0 |  | 0 |  |
| 2 | -1.115^***^ | (-3.86) | -1.137^***^ | (-3.87) | -1.134^***^ | (-3.91) | -1.081^***^ | (-3.77) |
| 3 | -2.378^***^ | (-4.06) | -2.396^***^ | (-4.14) | -2.321^***^ | (-3.91) | -2.289^***^ | (-3.88) |
| 4 | -2.389^***^ | (-4.08) | -2.398^***^ | (-4.04) | -2.427^***^ | (-4.25) | -2.400^***^ | (-4.06) |
| 5 or 6 | -3.998^***^ | (-4.37) | -3.970^***^ | (-4.31) | -4.060^***^ | (-4.32) | -3.936^***^ | (-4.27) |
| Age: 80-84 | 0 |  | 0 |  | 0 |  | 0 |  |
| 85-89 | -0.701^*^ | (-2.39) | -0.708^*^ | (-2.38) | -0.683^*^ | (-2.35) | -0.687^*^ | (-2.40) |
| 90+ | -0.739^*^ | (-2.31) | -0.833^**^ | (-2.61) | -0.779^*^ | (-2.56) | -0.761^*^ | (-2.43) |
| Male, not alone | 0 |  | 0 |  | 0 |  | 0 |  |
| Male, lives alone | -0.754^+^ | (-1.84) | -0.777^*^ | (-1.99) | -0.822^*^ | (-2.02) | -0.717^+^ | (-1.80) |
| Female, not alone | -0.096 | (-0.30) | 0.054 | (0.17) | 0.012 | (0.03) | -0.127 | (-0.40) |
| Female, lives alone | -0.541^+^ | (-1.66) | -0.345 | (-1.01) | -0.539^+^ | (-1.67) | -0.516 | (-1.57) |
| Has a child | -0.358 | (-1.07) | -0.355 | (-1.11) | -0.364 | (-1.14) | -0.252 | (-0.77) |
| Education: Low |  |  | 0 |  |  |  |  |  |
| Middle |  |  | 0.277 | (0.88) |  |  |  |  |
| High |  |  | 0.788^*^ | (2.33) |  |  |  |  |
| Homeowner |  |  | 0.295 | (1.08) |  |  |  |  |
| Income: <€1,500 |  |  |  |  | 0 |  |  |  |
| €1,500-1,999 |  |  |  |  | 0.496 | (1.48) |  |  |
| €2,000+ |  |  |  |  | 0.464 | (1.43) |  |  |
| Wealth: <€12,500 |  |  |  |  |  |  | 0 |  |
| €12,500-24,999 |  |  |  |  |  |  | -0.147 | (-0.34) |
| €25,000+ |  |  |  |  |  |  | 0.540^*^ | (2.07) |
| Constant | 1.232^**^ | (2.84) | 0.792^+^ | (1.65) | 1.020^*^ | (2.24) | 0.966^*^ | (2.17) |
| *N* | 573 |  |  |  |  |  |  |  |

Table A8: Care deficit in England: stepwise models

|  | (1) |  | (2) |  | (3) |  | (4) |  |
| --- | --- | --- | --- | --- | --- | --- | --- | --- |
|  | Coef. | t | Coef. | t | Coef. | t | Coef. | t |
|  |  |  |  |  |  |  |  |  |
| ADL needs: 1 | 0 |  | 0 |  | 0 |  | 0 |  |
| 2 | -0.925^**^ | (-3.11) | -0.927^**^ | (-3.08) | -0.925^**^ | (-3.10) | -0.870^**^ | (-2.90) |
| 3 | -1.037^**^ | (-2.72) | -0.999^**^ | (-2.63) | -1.034^**^ | (-2.70) | -1.126^**^ | (-2.89) |
| 4 | -2.435^***^ | (-4.68) | -2.422^***^ | (-4.83) | -2.432^***^ | (-4.69) | -2.422^***^ | (-4.48) |
| 5 or 6 | -2.221^***^ | (-4.08) | -2.209^***^ | (-4.01) | -2.219^***^ | (-4.06) | -2.201^***^ | (-4.04) |
| Age: 80-84 | 0 |  | 0 |  | 0 |  | 0 |  |
| 85-89 | -0.245 | (-0.86) | -0.289 | (-0.98) | -0.247 | (-0.87) | -0.208 | (-0.71) |
| 90+ | -1.128^***^ | (-3.47) | -1.110^***^ | (-3.36) | -1.127^***^ | (-3.42) | -1.173^***^ | (-3.65) |
| Male, not alone | 0 |  | 0 |  | 0 |  | 0 |  |
| Male, lives alone | 1.544^***^ | (3.95) | 1.722^***^ | (4.22) | 1.545^***^ | (3.95) | 1.658^***^ | (4.17) |
| Female, not alone | -0.114 | (-0.34) | 0.058 | (0.17) | -0.113 | (-0.34) | -0.006 | (-0.02) |
| Female, lives alone | 0.743^*^ | (2.39) | 0.955^**^ | (2.73) | 0.743^*^ | (2.40) | 0.963^**^ | (2.85) |
| Has a child | 0.163 | (0.42) | 0.188 | (0.50) | 0.164 | (0.43) | 0.310 | (0.79) |
| Education: Low |  |  | 0 |  |  |  |  |  |
| Middle |  |  | 0.194 | (0.73) |  |  |  |  |
| High |  |  | 0.884^*^ | (2.13) |  |  |  |  |
| Homeowner |  |  | 0.333 | (1.23) |  |  |  |  |
| Income: <€1,500 |  |  |  |  | 0 |  |  |  |
| €1,500-1,999 |  |  |  |  | -0.033 | (-0.11) |  |  |
| €2,000+ |  |  |  |  | 0.012 | (0.04) |  |  |
| Wealth: <€12,500 |  |  |  |  |  |  | 0 |  |
| €12,500-24,999 |  |  |  |  |  |  | -0.341 | (-0.87) |
| €25,000+ |  |  |  |  |  |  | 0.725^**^ | (2.71) |
| Constant | 0.123 | (0.28) | -0.423 | (-0.81) | 0.127 | (0.28) | -0.448 | (-0.88) |
| *N* | 395 |  |  |  |  |  |  |  |

Table A9: Models for Figure 3A – Income groups

|  | (1) |  | (2) |  | (3) |  | (4) |  | (5) |  | (6) |  |
| --- | --- | --- | --- | --- | --- | --- | --- | --- | --- | --- | --- | --- |
|  | Germany |  | England |  | Germany |  | England |  | Germany |  | England |  |
|  | Informal | t | Informal | t | Formal | t | Formal | t | Unmet n. | t | Unmet n. | t |
|  |  |  |  |  |  |  |  |  |  |  |  |  |
| ADL needs: 1 | 0 |  | 0 |  | 0 |  | 0 |  | 0 |  | 0 |  |
| 2 | 1.017^***^ | (3.71) | 0.931^**^ | (3.07) | 0.911^**^ | (3.30) | 0.799^+^ | (1.94) | -1.134^***^ | (-3.91) | -0.925^**^ | (-3.10) |
| 3 | 1.834^***^ | (5.04) | 1.250^**^ | (3.06) | 0.976^**^ | (3.01) | 0.579 | (1.10) | -2.321^***^ | (-3.91) | -1.034^**^ | (-2.70) |
| 4 | 1.954^***^ | (4.34) | 1.519^***^ | (3.34) | 1.897^***^ | (4.66) | 1.751^***^ | (3.70) | -2.427^***^ | (-4.25) | -2.432^***^ | (-4.69) |
| 5 | 2.954^***^ | (4.99) | 1.017^+^ | (1.81) | 1.740^***^ | (3.42) | 2.269^***^ | (3.38) | -4.060^***^ | (-4.32) | -2.219^***^ | (-4.06) |
| 6 | 4.751^***^ | (4.87) | 0.880^+^ | (1.66) | 1.413^**^ | (2.86) | 3.664^***^ | (6.29) |  |  |  |  |
| Age: 80-84 | 0 |  | 0 |  | 0 |  | 0 |  | 0 |  | 0 |  |
| 85-89 | 0.423 | (1.37) | 0.177 | (0.61) | 0.086 | (0.33) | -0.298 | (-0.77) | -0.683^*^ | (-2.35) | -0.247 | (-0.87) |
| 90+ | 0.626^*^ | (2.13) | 0.845^**^ | (2.64) | -0.129 | (-0.48) | 0.539 | (1.45) | -0.779^*^ | (-2.56) | -1.127^***^ | (-3.42) |
| Male, not alone | 0 |  | 0 |  | 0 |  | 0 |  | 0 |  | 0 |  |
| Male, lives alone | 0.031 | (0.08) | -1.676^***^ | (-4.22) | 0.917^*^ | (2.41) | 0.810^+^ | (1.80) | -0.822^*^ | (-2.02) | 1.545^***^ | (3.95) |
| Female, not alone | -0.291 | (-1.03) | -0.006 | (-0.02) | 0.189 | (0.55) | 0.253 | (0.41) | 0.012 | (0.03) | -0.113 | (-0.34) |
| Female, lives alone | -0.605^*^ | (-2.27) | -1.420^***^ | (-4.44) | 1.246^***^ | (4.57) | 1.703^***^ | (4.00) | -0.539^+^ | (-1.67) | 0.743^*^ | (2.40) |
| Has a child | 0.482 | (1.46) | 0.611 | (1.47) | -0.138 | (-0.48) | -0.855^*^ | (-2.07) | -0.364 | (-1.14) | 0.164 | (0.43) |
| Income: <€1,500 | 0 |  | 0 |  | 0 |  | 0 |  | 0 |  | 0 |  |
| €1,500-1,999 | -0.529 | (-1.64) | -0.266 | (-0.94) | -0.131 | (-0.47) | 0.474 | (1.22) | 0.496 | (1.48) | -0.033 | (-0.11) |
| €2,000+ | -0.720^*^ | (-2.46) | -0.124 | (-0.38) | -0.091 | (-0.33) | 0.304 | (0.75) | 0.464 | (1.43) | 0.012 | (0.04) |
| Constant | -1.217^**^ | (-2.63) | -0.771^+^ | (-1.65) | -1.597^***^ | (-4.01) | -2.690^***^ | (-4.96) | 1.020^*^ | (2.24) | 0.127 | (0.28) |
| *N* | 573 |  | 395 |  | 573 |  | 395 |  | 573 |  | 395 |  |

Table A10: Models for Figure 4A – wealth groups

|  | (1) |  | (2) |  | (3) |  | (4) |  | (5) |  | (6) |  |
| --- | --- | --- | --- | --- | --- | --- | --- | --- | --- | --- | --- | --- |
|  | Germany |  | England |  | Germany |  | England |  | Germany |  | England |  |
|  | Informal | t | Informal | t | Formal | t | Formal | t | Unmet n. | t | Unmet n. | t |
|  |  |  |  |  |  |  |  |  |  |  |  |  |
| ADL needs: 1 | 0 |  | 0 |  | 0 |  | 0 |  | 0 |  | 0 |  |
| 2 | 1.003^***^ | (3.67) | 0.888^**^ | (2.94) | 0.883^**^ | (3.20) | 0.746^+^ | (1.78) | -1.081^***^ | (-3.77) | -0.870^**^ | (-2.90) |
| 3 | 1.793^***^ | (4.98) | 1.232^**^ | (3.02) | 0.973^**^ | (3.08) | 0.651 | (1.23) | -2.289^***^ | (-3.88) | -1.126^**^ | (-2.89) |
| 4 | 1.936^***^ | (4.00) | 1.519^***^ | (3.37) | 1.932^***^ | (4.68) | 1.673^***^ | (3.34) | -2.400^***^ | (-4.06) | -2.422^***^ | (-4.48) |
| 5 | 2.908^***^ | (5.18) | 0.964^+^ | (1.77) | 1.769^***^ | (3.47) | 2.313^***^ | (3.56) | -3.936^***^ | (-4.27) | -2.201^***^ | (-4.04) |
| 6 | 4.553^***^ | (4.37) | 0.929^+^ | (1.73) | 1.395^**^ | (2.82) | 3.452^***^ | (6.07) |  |  |  |  |
| Age: 80-84 | 0 |  | 0 |  | 0 |  | 0 |  | 0 |  | 0 |  |
| 85-89 | 0.338 | (1.11) | 0.199 | (0.69) | 0.103 | (0.39) | -0.344 | (-0.89) | -0.687^*^ | (-2.40) | -0.208 | (-0.71) |
| 90+ | 0.632^*^ | (2.20) | 0.904^**^ | (2.79) | -0.158 | (-0.59) | 0.549 | (1.45) | -0.761^*^ | (-2.43) | -1.173^***^ | (-3.65) |
| Male, not alone | 0 |  | 0 |  | 0 |  | 0 |  | 0 |  | 0 |  |
| Male, lives alone | -0.214 | (-0.55) | -1.744^***^ | (-4.36) | 0.948^*^ | (2.44) | 0.786^+^ | (1.80) | -0.717^+^ | (-1.80) | 1.658^***^ | (4.17) |
| Female, not alone | -0.191 | (-0.64) | -0.030 | (-0.09) | 0.261 | (0.81) | 0.080 | (0.13) | -0.127 | (-0.40) | -0.006 | (-0.02) |
| Female, lives alone | -0.718^**^ | (-2.67) | -1.496^***^ | (-4.37) | 1.288^***^ | (4.87) | 1.499^***^ | (3.59) | -0.516 | (-1.57) | 0.963^**^ | (2.85) |
| Has child | 0.351 | (1.02) | 0.564 | (1.37) | -0.181 | (-0.60) | -0.962^*^ | (-2.29) | -0.252 | (-0.77) | 0.310 | (0.79) |
| Wealth: <€12,500 | 0 |  | 0 |  | 0 |  | 0 |  | 0 |  | 0 |  |
| €12,500-24,999 | -0.679^+^ | (-1.78) | -0.325 | (-0.84) | 0.550 | (1.55) | 0.248 | (0.50) | -0.147 | (-0.34) | -0.341 | (-0.87) |
| €25,000+ | -1.234^***^ | (-4.82) | -0.361 | (-1.31) | -0.021 | (-0.10) | -0.608^+^ | (-1.71) | 0.540^*^ | (2.07) | 0.725^**^ | (2.71) |
| Constant | -0.870^+^ | (-1.82) | -0.564 | (-1.14) | -1.690^***^ | (-4.18) | -2.065^***^ | (-3.41) | 0.966^*^ | (2.17) | -0.448 | (-0.88) |
| *N* | 573 |  | 395 |  | 573 |  | 395 |  | 573 |  | 395 |  |

Table A11: Models for Figure 2B – Interaction of education with number of care needs

|  | (1) |  | (2) |  | (3) |  | (4) |  | (5) |  | (6) |  |
| --- | --- | --- | --- | --- | --- | --- | --- | --- | --- | --- | --- | --- |
|  | Germany |  | England | | Germany | | England | | Germany | | England | |
|  | Informal | t | Informal |  | Formal | t | Formal | t | Unmet n | t | Unmet n | t |
|  |  |  |  |  |  |  |  |  |  |  |  |  |
| Low educ. & 1-2 ADL | 0 |  | 0 |  | 0 |  | 0 |  | 0 |  | 0 |  |
| 3-6 ADL | 1.902^***^ | (4.50) | 0.547 | (1.45) | 0.703^*^ | (2.08) | 1.676^***^ | (4.20) | -2.52^***^ | (-4.00) | -1.51^***^ | (-3.43) |
| Mid educ. & 1-2 ADL | -0.373 | (-1.15) | -0.371 | (-1.28) | -0.035 | (-0.12) | 0.163 | (0.39) | 0.209 | (0.69) | 0.232 | (0.81) |
| 3-6 ADL | 1.457^***^ | (4.17) | 0.651^+^ | (1.68) | 1.004^**^ | (2.63) | 1.307^**^ | (2.82) | -1.831^**^ | (-3.24) | -0.933^*^ | (-2.38) |
| High educ, & 1-2 ADL | -0.540 | (-1.59) | -1.333^*^ | (-2.39) | -0.697 | (-1.50) | -0.573 | (-0.70) | 0.880^*^ | (2.18) | 1.127^*^ | (2.23) |
| 3-6 ADL | 0.840^+^ | (1.72) | 0.595 | (0.95) | 1.357^**^ | (3.08) | 2.946^***^ | (4.21) | -2.235^**^ | (-2.69) | -1.394^+^ | (-1.68) |
| Age: 80-84 | 0 |  | 0 |  | 0 |  | 0 |  | 0 |  | 0 |  |
| 85-89 | 0.498^+^ | (1.67) | 0.149 | (0.52) | 0.112 | (0.42) | -0.295 | (-0.76) | -0.735^*^ | (-2.56) | -0.224 | (-0.78) |
| 90+ | 0.717^*^ | (2.47) | 0.684^*^ | (2.18) | -0.091 | (-0.35) | 0.748^*^ | (2.17) | -0.892^**^ | (-2.87) | -1.040^**^ | (-3.25) |
| Male, not alone | 0 |  | 0 |  | 0 |  | 0 |  | 0 |  | 0 |  |
| Male, lives alone | -0.123 | (-0.32) | -1.67^***^ | (-4.28) | 0.930^*^ | (2.58) | 0.733 | (1.56) | -0.694^+^ | (-1.81) | 1.513^***^ | (3.86) |
| Female, not alone | -0.237 | (-0.88) | -0.174 | (-0.53) | 0.274 | (0.86) | 0.143 | (0.28) | 0.040 | (0.13) | 0.075 | (0.22) |
| Female, lives alone | -0.847^**^ | (-3.30) | -1.36^***^ | (-4.20) | 1.141^***^ | (4.00) | 1.401^***^ | (3.53) | -0.288 | (-0.91) | 0.802^*^ | (2.45) |
| Has child | 0.373 | (1.19) | 0.591 | (1.48) | -0.159 | (-0.56) | -0.714^+^ | (-1.75) | -0.276 | (-0.93) | 0.116 | (0.30) |
| Constant | -0.608 | (-1.46) | -0.181 | (-0.37) | -1.126^**^ | (-2.61) | -2.24^***^ | (-3.73) | 0.410 | (0.97) | -0.419 | (-0.83) |
| *N* | 573 |  | 395 |  | 573 |  | 395 |  | 573 |  | 395 |  |

Table A12: Models for Figure 3B – Interaction of income with number of care needs

|  | (1) |  | (2) |  | (3) |  | (4) |  | (5) |  | (6) |  |
| --- | --- | --- | --- | --- | --- | --- | --- | --- | --- | --- | --- | --- |
|  | Germany |  | England |  | Germany |  | England |  | Germany |  | England |  |
|  | Informal | t | Informal | t | Formal | t | Formal | t | Unmet n. | t | Unmet n. | t |
|  |  |  |  |  |  |  |  |  |  |  |  |  |
| Low income & 1-2 ADL | 0 |  | 0 |  | 0 |  | 0 |  | 0 |  | 0 |  |
| 3-6 ADL | 1.883^***^ | (5.41) | 0.779^*^ | (2.28) | 0.610^*^ | (2.39) | 1.872^***^ | (4.84) | -1.961^***^ | (-3.81) | -1.405^***^ | (-3.89) |
| Mid income & 1-2 ADL | -0.168 | (-0.48) | -0.332 | (-0.95) | -0.378 | (-1.08) | 0.744 | (1.61) | 0.417 | (1.24) | 0.078 | (0.23) |
| 3-6 ADL | 1.216^**^ | (2.62) | 0.785^+^ | (1.69) | 1.125^*^ | (2.41) | 1.399^**^ | (2.73) | -2.112^***^ | (-3.53) | -1.716^***^ | (-3.31) |
| High income & 1-2 ADL | -0.774^*^ | (-2.06) | -0.100 | (-0.26) | -0.532^+^ | (-1.75) | -0.012 | (-0.02) | 0.689^*^ | (1.99) | -0.039 | (-0.11) |
| 3-6 ADL | 1.224^**^ | (3.04) | 0.624 | (1.28) | 1.352^**^ | (3.12) | 1.979^**^ | (3.25) | -2.959^**^ | (-2.77) | -1.046^+^ | (-1.69) |
| Age: 80-84 | 0 |  | 0 |  | 0 |  | 0 |  | 0 |  | 0 |  |
| 85-89 | 0.460 | (1.52) | 0.157 | (0.57) | 0.108 | (0.41) | -0.252 | (-0.65) | -0.711^*^ | (-2.51) | -0.221 | (-0.79) |
| 90+ | 0.700^*^ | (2.46) | 0.753^*^ | (2.37) | -0.104 | (-0.40) | 0.717^*^ | (2.10) | -0.859^**^ | (-2.86) | -1.100^***^ | (-3.43) |
| Male, not alone | 0 |  | 0 |  | 0 |  | 0 |  | 0 |  | 0 |  |
| Male, lives alone | -0.057 | (-0.14) | -1.566^***^ | (-4.09) | 0.941^*^ | (2.55) | 0.675 | (1.50) | -0.754^+^ | (-1.83) | 1.419^***^ | (3.75) |
| Female, not alone | -0.228 | (-0.82) | -0.033 | (-0.10) | 0.248 | (0.76) | 0.093 | (0.18) | -0.027 | (-0.09) | -0.079 | (-0.24) |
| Female, lives alone | -0.751^**^ | (-2.96) | -1.223^***^ | (-4.00) | 1.177^***^ | (4.20) | 1.301^***^ | (3.43) | -0.419 | (-1.38) | 0.654^*^ | (2.19) |
| Has child | 0.411 | (1.29) | 0.657 | (1.64) | -0.144 | (-0.49) | -0.801^+^ | (-1.96) | -0.326 | (-1.05) | 0.090 | (0.24) |
| Constant | -0.740^+^ | (-1.72) | -0.523 | (-1.12) | -1.050^**^ | (-2.79) | -2.251^***^ | (-4.03) | 0.493 | (1.15) | -0.104 | (-0.22) |
| *N* | 573 |  | 395 |  | 573 |  | 395 |  | 573 |  | 395 |  |

Table A13: Models for Figure 4B – Interaction of wealth with number of care needs

|  | (1) |  | (2) |  | (3) |  | (4) |  | (5) |  | (6) |  |
| --- | --- | --- | --- | --- | --- | --- | --- | --- | --- | --- | --- | --- |
|  | Germany |  | England |  | Germany |  | England |  | Germany |  | England |  |
|  | Informal | t | Informal | t | Formal | t | Formal | t | Unmet n. | t | Unmet n. | t |
|  |  |  |  |  |  |  |  |  |  |  |  |  |
| Wealth <€25k & 1-2 ADL | 0 |  | 0 |  | 0 |  | 0 |  | 0 |  | 0 |  |
| 3-6 ADL | 1.868^***^ | (6.01) | 0.705^+^ | (1.92) | 0.795^**^ | (3.03) | 1.395^***^ | (3.70) | -2.112^***^ | (-4.43) | -1.390^***^ | (-3.51) |
| Wealth €25k+ & 1-2 ADL | -1.001^**^ | (-3.09) | -0.413 | (-1.43) | -0.419 | (-1.60) | -1.120^*^ | (-2.51) | 0.714^*^ | (2.37) | 0.917^**^ | (3.25) |
| 3-6 ADL | 0.489 | (1.35) | 0.580 | (1.62) | 1.272^***^ | (3.61) | 0.889^*^ | (2.11) | -2.035^***^ | (-3.55) | -0.630 | (-1.60) |
| Age: 80-84 | 0 |  | 0 |  | 0 |  | 0 |  | 0 |  | 0 |  |
| 85-89 | 0.414 | (1.41) | 0.157 | (0.57) | 0.117 | (0.44) | -0.292 | (-0.77) | -0.718^**^ | (-2.59) | -0.200 | (-0.71) |
| 90+ | 0.681^*^ | (2.40) | 0.768^*^ | (2.44) | -0.097 | (-0.38) | 0.772^*^ | (2.24) | -0.859^**^ | (-2.87) | -1.169^***^ | (-3.76) |
| Male, not alone | 0 |  | 0 |  | 0 |  | 0 |  | 0 |  | 0 |  |
| Male, lives alone | -0.240 | (-0.64) | -1.607^***^ | (-4.20) | 0.849^*^ | (2.34) | 0.555 | (1.22) | -0.593 | (-1.55) | 1.547^***^ | (4.04) |
| Female, not alone | -0.103 | (-0.39) | -0.080 | (-0.25) | 0.265 | (0.82) | -0.005 | (-0.01) | -0.164 | (-0.55) | 0.037 | (0.11) |
| Female, lives alone | -0.795^**^ | (-3.14) | -1.315^***^ | (-4.09) | 1.115^***^ | (4.10) | 1.138^**^ | (2.95) | -0.362 | (-1.20) | 0.919^**^ | (2.85) |
| Has child | 0.252 | (0.77) | 0.593 | (1.45) | -0.178 | (-0.59) | -0.994^*^ | (-2.33) | -0.217 | (-0.68) | 0.289 | (0.76) |
| Constant | -0.495 | (-1.15) | -0.311 | (-0.62) | -1.063^**^ | (-2.66) | -1.376^*^ | (-2.34) | 0.416 | (0.96) | -0.835 | (-1.64) |
| *N* | 573 |  | 395 |  | 573 |  | 395 |  | 573 |  | 395 |  |

Table A14: Significance tests for SES differences of the estimated marginal probabilities: Comparing the estimated probabilities between the high- and the low-SES groups

|  | Informal care | | Formal care | | Unmet needs | |
| --- | --- | --- | --- | --- | --- | --- |
|  | Germany | England | Germany | England | Germany | England |
| Hypotheses: |  | |  | |  | |
| Low-intensity care needs | H1: Prob. for low SES > Prob. for high SES | | H2: Prob. for low SES = Prob. for high SES | | H3: Prob. for low SES < Prob. for high SES | |
| High-intensity care needs | H4: Prob. for low SES > Prob. for high SES | | H5: Prob. for low SES < Prob. for high SES | | H6: Prob. for low SES = Prob. for high SES | |
| Null Hypotheses: |  |  |  |  |  |  |
| Low intensity | Low<=high | Low<=high | Low=high | Low=high | Low>=high | Low>=high |
| High intensity | Low<=high | Low<=high | Low>=high | Low>=high | Low=high | Low=high |
|  |  |  |  |  |  |  |
| Education |  |  |  |  |  |  |
| Low intensity | † | ** | n.s. | n.s.. | * | ** |
| High intensity | * | n.s. | * | * | n.s. | n.s. |
|  |  |  |  |  |  |  |
| Income |  |  |  |  |  |  |
| Low intensity | * | n.s. | † | n.s. | * | n.s. |
| High intensity | † | n.s. | * | n.s. | n.s. | n.s. |
|  |  |  |  |  |  |  |
| Wealth |  |  |  |  |  |  |
| Low intensity | ** | † | † | ** | * | ** |
| High intensity | ** | n.s. | † | n.s. | n.s. | † |

Notes: † p<0.1, * p<0.05, ** p<0.01

The tests refer to the estimated margins displayed in Figures 2B, 3B and 4B.
